# Histotripsy Ablation of Spontaneously Occurring Canine Bone Tumors *In Vivo*

**DOI:** 10.1109/TBME.2022.3191069

**Published:** 2022-07-14

**Authors:** Lauren N. Ruger, Alayna N. Hay, Jessica M. Gannon, Hannah O. Sheppard, Sheryl L. Coutermarsh-Ott, Gregory B. Daniel, Katharine R. Kierski, Brittany J. Ciepluch, Eli Vlaisavljevich, Joanne L. Tuohy

**Affiliations:** Department of Biomedical Engineering and Mechanics, Vir Polytechnic Institute and State University, USA.; Department of Small Animal Clinical Sciences, Virginia-Maryland Regional College of Veterinary Medicine, USA; Virginia Tech Animal Cancer Care and Research Center, Virginia-Maryland Regional College of Veterinary Medicine, USA.; Department of Biomedical Engineering and Mechanics, Vir Polytechnic Institute and State University, USA.; Department of Biomedical Engineering and Mechanics, Vir Polytechnic Institute and State University, USA.; Department of Biological Sciences and Pathobiology, Virginia Polytechnic Institute and State University, USA.; Department of Small Animal Clinical Sciences, Virginia-Maryland Regional College of Veterinary Medicine, USA; Virginia Tech Animal Cancer Care and Research Center, Virginia-Maryland Regional College of Veterinary Medicine, USA.; Department of Small Animal Clinical Sciences, Virginia-Maryland Regional College of Veterinary Medicine, USA; Virginia Tech Animal Cancer Care and Research Center, Virginia-Maryland Regional College of Veterinary Medicine, USA.; Department of Small Animal Clinical Sciences, Virginia-Maryland Regional College of Veterinary Medicine, USA; Virginia Tech Animal Cancer Care and Research Center, Virginia-Maryland Regional College of Veterinary Medicine, USA.; Department of Biomedical Engineering and Mechanics, Vir Polytechnic Institute and State University, Blacksburg, VA 24061 USA.; Department of Small Animal Clinical Sciences, Virginia-Maryland Regional College of Veterinary Medicine, Blacksburg, VA 24061 USA; Virginia Tech Animal Cancer Care and Research Center, Virginia-Maryland Regional College of Veterinary Medicine, Roanoke, VA 24016 USA

**Keywords:** Ablation, bone tumors, canine, focused ultrasound, histotripsy, osteosarcoma

## Abstract

**Objective:**

Osteosarcoma (OS) is a devastating primary bone tumor in dogs and humans with limited non-surgical treatment options. As the first completely non-invasive and non-thermal ablation technique, histotripsy has the potential to significantly improve the standard of care for patients with primary bone tumors.

**Introduction:**

Standard of care treatment for primary appendicular OS involves surgical resection via either limb amputation or limb-salvage surgery for suitable candidates. Biological similarities between canine and human OS make the dog an informative comparative oncology research model to advance treatment options for primary OS. Evaluating histotripsy for ablating spontaneous canine primary OS will build a foundation upon which histotripsy can be translated clinically into a standard of care therapy for canine and human OS.

**Methods:**

Five dogs with suspected spontaneous OS were treated with a 500 kHz histotripsy system guided by real-time ultrasound image guidance. Spherical ablation volumes within each tumor (1.25–3 cm in diameter) were treated with single cycle histotripsy pulses applied at a pulse repetition frequency of 500 Hz and a dose of 500 pulses/point.

**Results:**

Tumor ablation was successfully identified grossly and histologically within the targeted treatment regions of all subjects. Histotripsy treatments were well-tolerated amongst all patients with no significant clinical adverse effects.

**Conclusion & Significance:**

Histotripsy safely and effectively ablated the targeted treatment volumes in all subjects, demonstrating its potential to serve as a non-invasive treatment modality for primary bone tumors.

## I. Introduction

Osteosarcoma (OS) is the most common primary bone tumor in both humans and canines, primarily affecting adolescent children with a worldwide incidence of approximately three to four cases per 1 million people in this age group [1]. The prevalence of OS is even greater in canines with ~13.9 cases per 100000 dogs [2]. For both human and canine OS patients, the prognosis is grim, with a 5-year survival rate of only 60 to 70% in human patients [3] and a 10 to 12 month median survival in canine patients [2]. Canine OS shares numerous biological similarities with human OS, allowing the dog to serve as a valuable comparative oncology research model. Current standard of care treatment for primary appendicular OS consists of limb amputation or limb salvage surgery for both canines and humans [4]. Not all patients are suitable candidates for a limb amputation due to factors such as concurrent debilitating orthopedic or neurologic disease or the desire to avoid amputation. Limb salvage surgery is associated with a risk of complications in dogs and people. In dogs, limb salvage surgery is limited to select anatomic sites such as the distal radius, and reported complications include deep surgical site infections and implant failure [5]. In humans, advances in surgical techniques have reduced the risk of complications associated with limb salvage surgery, but risks remain, including infection, implant failure, and graft fractures [6], [7]. Therefore, novel limb salvage techniques, especially non-invasive techniques, are needed to advance treatment options for appendicular OS patients.

The innovative focused ultrasound technique, histotripsy, has the potential to serve as a non-invasive, novel limb salvage technique for treating OS. Histotripsy is a precise, non-thermal, non-invasive, and non-ionizing ablation technique which generates acoustic cavitation bubble clouds within a target tissue using high amplitude focused ultrasound pulses to mechanically homogenize the tissue [8]–[10]. Previously, thermal ablation techniques, including radiofrequency [11], microwave ablation [12], laser therapy [13], and high intensity focused ultrasound (HIFU) [14], have been applied as palliative treatment methods and/or ablation methods for the treatment of primary and metastatic bone tumors. Thermal modalities, however, pose the risk of complications such as soft tissue burns or damage to surrounding healthy tissues such as nerves and bone [15]–[17]. Histotripsy non-thermally generates acoustic cavitation to mechanically destroy targeted tissue and exhibits tissue selective properties [18]–[20], potentially allowing it to overcome these challenges. Additionally, the cavitation bubble cloud formed during histotripsy treatment and the resulting tissue ablation can be visualized on ultrasound imaging and magnetic resonance imaging (MRI), allowing real-time monitoring of treatment delivery and tissue disintegration. Together, these features allow histotripsy to be utilized for precise, non-invasive tissue ablation that has shown promise for multiple applications. Histotripsy has previously been employed to successfully ablate numerous tumor types *in vivo* in preclinical models including prostate [21], kidney [22], and liver tumors [23]. In addition, initial human clinical trial results have shown safe and effective ablation of primary and metastatic liver tumors [24].

To build on this prior work, our group recently conducted a study demonstrating the feasibility of ablating *ex vivo* bone tumors collected from canine OS patients [25]. In this *ex vivo* study, sectioned tumor samples were treated using a 500 kHz transducer and a treatment dosage of 4000 pulses per treatment point. Results revealed fully ablated tissue within the targeted regions of the tumor, with no damage to overlying tissues. Additionally, no significant changes were observed when healthy bone and nerve samples were treated with the same histotripsy parameters. These results suggest that histotripsy can safely be employed to treat primary bone tumors, including those located in regions near critical structures.

The objective of the current study was to determine the *in vivo* safety and feasibility of ablating primary bone tumors with histotripsy in canine OS patients. To test this, a prototype histotripsy system equipped with a custom 500 kHz histotripsy transducer was used to treat five client-owned dogs with suspected OS enrolled in an IACUC-approved veterinary clinical trial. The safety of histotripsy was determined based on the incidence of adverse effects after histotripsy, and treatment effectiveness was assessed post-treatment via contrast enhanced computed tomography (CT) and histological analysis.

## II. Materials and Methods

### A. Animal and Study Approval

**Fig. 1.**
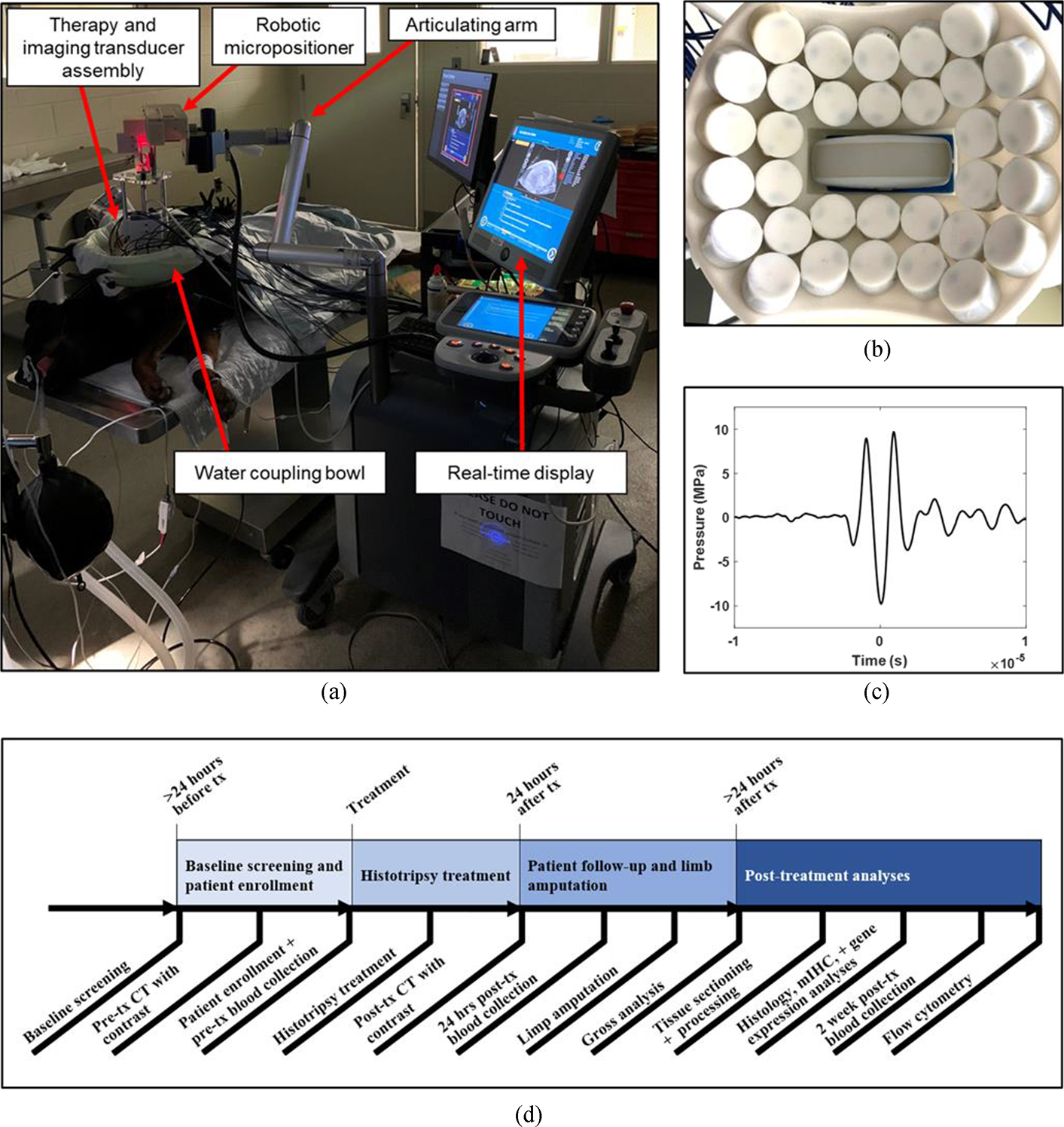
Clinical prototype histotripsy system and experimental timeline. (a) Image of the histotripsy system utilized in the current study for the treatment of primary bone tumors. A custom prototype 500 kHz histotripsy transducer with coaxially aligned ultrasound imaging probe was attached to the clinical prototype system with an articulating arm. The transducer assembly was guided via a motorized micropositioner and custom software to carry out the intended volumetric histotripsy treatment for each patient. (b) Image of the 500 kHz histotripsy transducer with coaxially aligned ultrasound imaging probe for real-time treatment feedback and monitoring. (c) Representative pressure waveform from the 500 kHz transducer used in this study. (d) Steps and timing of histotripsy study.

The experimental workflow and timeline followed in this study is summarized in Fig. 1(d). Client-owned dogs with appendicular bone lesions (n = 5) were enrolled in a clinical trial under an approved Virginia Tech Institutional Animal Care and Use Committee protocol (IACUC protocol number 19–229). Study enrollment criteria included: 1) dogs with a suspected diagnosis of appendicular OS based on cytologic or histologic evaluation of their bone lesion, 2) dogs that had not received any tumor-directed therapy or immunomodulatory drugs prior to study enrollment, and 3) dogs with no evidence of metastatic pulmonary lesions based on 3-view thoracic radiographs interpreted by a board-certified veterinary radiologist. Signed owner consent was obtained for enrollment of their pet into the clinical trial.

### B. Computed Tomography Imaging

CT scans with contrast of the affected limb were obtained using a Siemens Confidence RT multi-slice CT scanner prior to histotripsy treatment and at 18–24 hours after histotripsy treatment to characterize the CT appearance of histotripsy ablation of the tumor. CT tumor size measurements are reported as cross-sectional measurements based on maximal diameter on multiplanar reformation images. All CT images were assessed by a single board-certified veterinary radiologist (G.D.).

### C. Histotripsy System and Pressure Calibration

In this study, a 32-element 500 kHz histotripsy transducer was used to test the feasibility of histotripsy ablation for primary bone tumors in canine patients [Fig. 1]. The transducer array was organized around a central cut-out in the transducer designed to permit the coaxial alignment of a 3 MHz curvilinear ultrasonic imaging probe (Model C5-2, Analogic Corp., Peabody, MA, USA). The transducer had a geometric focus of 78 mm with elevational and transverse aperture sizes of 112 mm and 128 mm, respectively, and corresponding f-numbers of 0.70 (elevational) and 0.61 (transverse).

The histotripsy transducer was integrated onto a prototype clinical system (HistoSonics, Ann Arbor, MI, USA) before treatment by attaching the transducer and aligned imaging probe assembly to a triaxial robotic micro-positioner mounted on the clinical system using an articulating arm. The transducer was driven using a custom high-voltage pulser designed to generate short, single cycle therapy pulses and controlled by a preprogrammed field-programmable gate array (FPGA) board (Altera DE0-Nano, Terasic Technology, Dover, DE, USA). The transducer was powered by a high voltage DC power supply (GENH750W, TDK-Lambda, National City, CA, USA) and controlled by a custom MATLAB script (The MathWorks, Natick, MA, USA) written to receive a trigger from the clinical system. The coaxially aligned ultrasound probe was used for real-time treatment guidance and monitoring.

Before treatment, focal pressure waveforms for the histotripsy transducer were measured in degassed water at the transducer’s focal point using a high-sensitivity reference rod hydrophone (HNR-0500, Onda Corp., Sunnyvale, CA, USA) and a cross-calibrated custom-built fiber optic probe hydrophone (FOPH) [26], [27]. The rod hydrophone was also used to measure the lateral, elevational, and axial 1-D beam profiles of the transducer at a focal peak negative pressure (*p−*) of ~1.8 MPa by scanning the hydrophone incrementally over a distance wider than the focal width. The measured transverse, elevational, and axial full-width half-maximum (FWHM) dimensions at the geometric focus of the transducer were measured to be 2.1 mm, 2.1 mm, and 6.6 mm at the applied *p−*, respectively. Focal pressures were measured directly with the FOPH up to a peak negative pressure of ~20 MPa; at peak negative pressures greater than ~20 MPa, the focal pressure was estimated by summing measurements from a subset of a quarter and a half of the total elements to prevent cavitation from forming on the fiber as validated in previous studies [28]. All waveforms were measured using a Tektronix TBS2000 series oscilloscope at a sample rate of 500MS/s; then, the waveform data were averaged over 128 pulses and recorded in MATLAB.

### D. Histotripsy Treatment

Patient-specific treatment plans were developed using the pre-treatment CT images of the tumor, which provided guidance on the optimal area within the tumor to target for histotripsy treatment. Ideal histotripsy treatment areas were areas of bone lysis and/or tumor proliferation. The dogs were placed under general anesthesia maintained with inhaled isoflurane for their histotripsy ablations. Vital signs (temperature, heart rate, respiratory rate, blood pressure), ECG waveforms, and oxygen saturation levels (SpO2) were monitored and maintained by a licensed veterinary technician with veterinarian oversight throughout treatment. In preparation for histotripsy treatment, the hair overlying the planned treatment site was closely shaved using a combination of hair clippers and a shaving razor. Freehand ultrasound imaging was performed to determine the region of the bone tumor with the optimal treatment window (i.e., the area of bone lysis). The histotripsy transducer was then positioned over the treatment site using the articulating arm and placed in a container of degassed water (<30% dissolved O2) directly coupled to the canine patient using a surgical drape to allow for acoustic propagation from the transducer to the skin [Fig. 1(a)]. Fine adjustments to correctly position the transducer over the target region were made using the robotic micro-positioner. Prior to the volumetric tumor treatment, the pressure at the focus was increased incrementally until a visible bubble cloud was generated on ultrasound imaging or visible prefocal cavitation at the skin’s surface was noted to be significant. Then, a spherical treatment volume fully contained within the tumor was set manually within the software of the clinical histotripsy system. Treatment volumes ranged from 1.02 cm^3^ to 14.14 cm^3^ depending on total tumor size and accessibility. After identifying a pressure level for treatment and setting the treatment boundaries, an automated volumetric histotripsy treatment was applied to a 3D grid of equidistant treatment points within the defined boundaries. Treatment points were spaced by 3.5 mm in the axial direction and 1.5 mm in the lateral and elevational directions to allow overlap between the cavitation bubble cloud at each location. The integrated robotic micro-positioner was used to move the transducer focus between treatment locations, and approximately 500 histotripsy pulses were applied to each treatment point. The bubble cloud and tissue effects were monitored during treatment using real time ultrasound imaging. For some of the treatments, passive cavitation detection (PCD) was also used to monitor cavitation activity at the transducer focus. For the PCD monitoring, one of the transducer’s therapy elements was connected to an oscilloscope with a high voltage probe and used to detect the presence of cavitation in the focal region. To determine whether cavitation occurred during histotripsy pulsing, the signal generated by the backscattering of the incident pulse from the focus was monitored, similar to approaches used in previous histotripsy studies [29], [30]. The backscattered pressure amplitude was received by the PCD at the time point corresponding to approximately two times the time of flight for the focal length of the 500 kHz histotripsy transducer. Prefocal cavitation at the skin’s surface could also be monitored using the PCD signal, occurring at an earlier time point than the focal cavitation signal [29].

### E. Adverse Event Reporting

Any adverse events associated with histotripsy tumor ablation were graded using the Veterinary Cooperative Oncology Group Common Terminology Criteria for Adverse Events (VCOG-CTCAEv2) [31].

### F. Surgical Tumor Resection

At 18–24 hours after histotripsy treatment, the dogs were placed under general anesthesia maintained with inhaled isoflurane and underwent standard-of-care limb amputation surgery for resection of the primary tumor. Surgery was performed by board-certified veterinary surgical oncologists (J.T. and B.C.). The dogs were recovered in the intensive care unit after surgery and discharged to the care of their owners when deemed appropriate by the attending clinician.

### G. Gross and Microscopic Evaluation

Following limb amputation, each tumor was grossly evaluated by a single board-certified veterinary pathologist (S.C.O.) to identify the ablation zone and note the gross characteristics of histotripsy ablation. When possible, the study investigator (J.T.) was present at gross sectioning to confirm the location of the targeted volume within the tumor (e.g., depth, medial vs. lateral). If not, treatment areas were identified with inked marks on the skin or by detailed images sent prior to sectioning. Treated and untreated portions of the tumor were then sampled, fixed in 10% neutral buffered formalin and paraffin embedded according to routine protocols. Blocks were sectioned at 5 μM thickness and stained with hematoxylin and eosin using an automated stainer to determine the efficacy of the histotripsy ablation. For three of five patients, skin samples from the histotripsy treatment path were collected, processed, and analyzed for off-target histotripsy damage.

### H. Immune Response Evaluation

Peripheral blood samples of 10mLs were collected at the time of patient enrollment, 24 hours post (before amputation), and 2 weeks post histotripsy treatment into EDTA vacutainers via venipuncture. Blood samples were also collected from healthy control dogs matched to patients’ age and weight. Peripheral blood mononuclear cells (PMBCs) were isolated via density gradient separation, and 1×10^6^ PMBCs were stained for analysis via flow cytometry with the following antibodies: anti CD11b (clone M1/70), anti CD14 (clone MSE2), anti-CD80 (Clone 16-10A1), anti-CD4 (cloneYKIX302.9), and anti-CD62L (clone FMC46). The antibodies CD11b, CD14, and CD80 were purchased from BioLegend (San Diego, CA, USA) and CD4 and CD62L were purchased from BioRad Laboratories (Hercules, CA, USA). For staining, 1 × 10^6^ cells were incubated at 4 °C for 20 mins with canine Fc Inhibitor (Thermo Fisher Scientific, Waltham, MA) before the addition of primary antibodies. The PBMCs were incubated with primary antibodies for 30 mins at 4 °C, washed with FACS buffer (1x DPBS + 2.5% FBS) and stained with eFluor 506 viability dye (Thermo Fisher Scientific) following the manufacturer’s protocol. Samples were analyzed on a Cytoflex (Beckman Coulter, Brea, CA, USA) flow cytometer using acquisition software CytExpert and analysis software Kaluza. For analysis, lymphocyte or monocyte populations were gated based on forward and side scatter; then, single live cell populations were analyzed for the following phenotypes: CD4+ T- lymphocytes, CD4+ CD62L+ T- lymphocytes, CD11b+CD14+CD80+ monocytes, and CD11b+CD14+ CD62L+ monocytes.

For chromogenic multiplex immunohistochemistry (IHC), 5μM histotripsy-treated and untreated tumor tissue sections were stained with the following antibodies using a Roche Ventana Discovery Ultra Automated Research Stainer (Roche Diagnostics, Indianapolis, IN): IBA1 (FujiFilm, 019-19741, 1:300), iNOS (Abcam, ab3523, 1:100), CD206 (NovusBio, NBP190020, 1:200), CS4, (Origene, TA500477, 1:100), CD8 (Invitrogen, PAS-16893, 1:100, CD79a (Santa Cruz, sc-20064, 1:50), CD3 (DAKO, A0452, 1:1:100). Five archived OS tumor samples from dogs previously diagnosed with OS and not treated with histotripsy (Virginia Maryland College of Veterinary Medicine patients) were also evaluated. All sections were qualitatively evaluated by a board-certified veterinary pathologist (S.C.O.) for positive staining of the listed markers in histotripsy-treated and untreated areas. The markers selected for flow cytometry and IHC were chosen based on the availability of canine-specific reagents and our goal of providing a general evaluation of the immune response. We specifically evaluated monocytes and macrophages because they have previously been associated with improved OS patient prognosis [32], [33]. Lymphocytes also play an important role in modulating the immunosuppressive tumor microenvironment in OS [34], [35].For gene expression analysis, total RNA was extracted from 20μm formalin fixed paraffin embedded (FFPE) tissue scrolls sectioned from untreated and treated regions of patient tumor samples with the Zymo Quick-RNA FFPE extraction kit (Zymo, California, USA). The manufacturer’s protocol was followed (Zymo, California, USA). RNA quality and quantity were assessed with a NanoDrop One^C^ (Thermo Fisher), and only samples for patients with 260/280 ratio of 1.8–2.0 were used (n = 2 patients). The Qiagen RT^2^ First Strand kit was utilized to synthesize 400ng RNA to cDNA in accordance with the manufacturer’s protocol (Qiagen, Hilden, Germany). A custom 96 well RT^2^ PCR Canine Cancer Immune and Inflammation crosstalk array (Qiagen, Hilden, Germany) was utilized for differential gene expression analysis. Each array contained 89 genes relating to inflammation, immunity, and cancer, 3 housekeeping genes, and 4 internal quality control samples to evaluate genomic DNA contamination, reverse transcriptase, and assay efficiency (see Supplemental Table I for full gene list). Arrays were analyzed with an Applied Biosystems 7500 Fast Real-Time PCR System (Thermo Fisher). For data analysis, the cycle threshold (Ct) values acquired from each array were analyzed using the GeneGlobe RT^2^ Profiler PCR Data Analysis tool (ΔΔCt method) (Qiagen). The arithmetic means of the house keeping genes *HPRT1*, *GAPDH,* and *ACTB* were used for normalization, and gene expression changes in treated samples were calculated relative to untreated regions of the tumor.

## III. Results

### A. Study Population

**Fig. 2.**
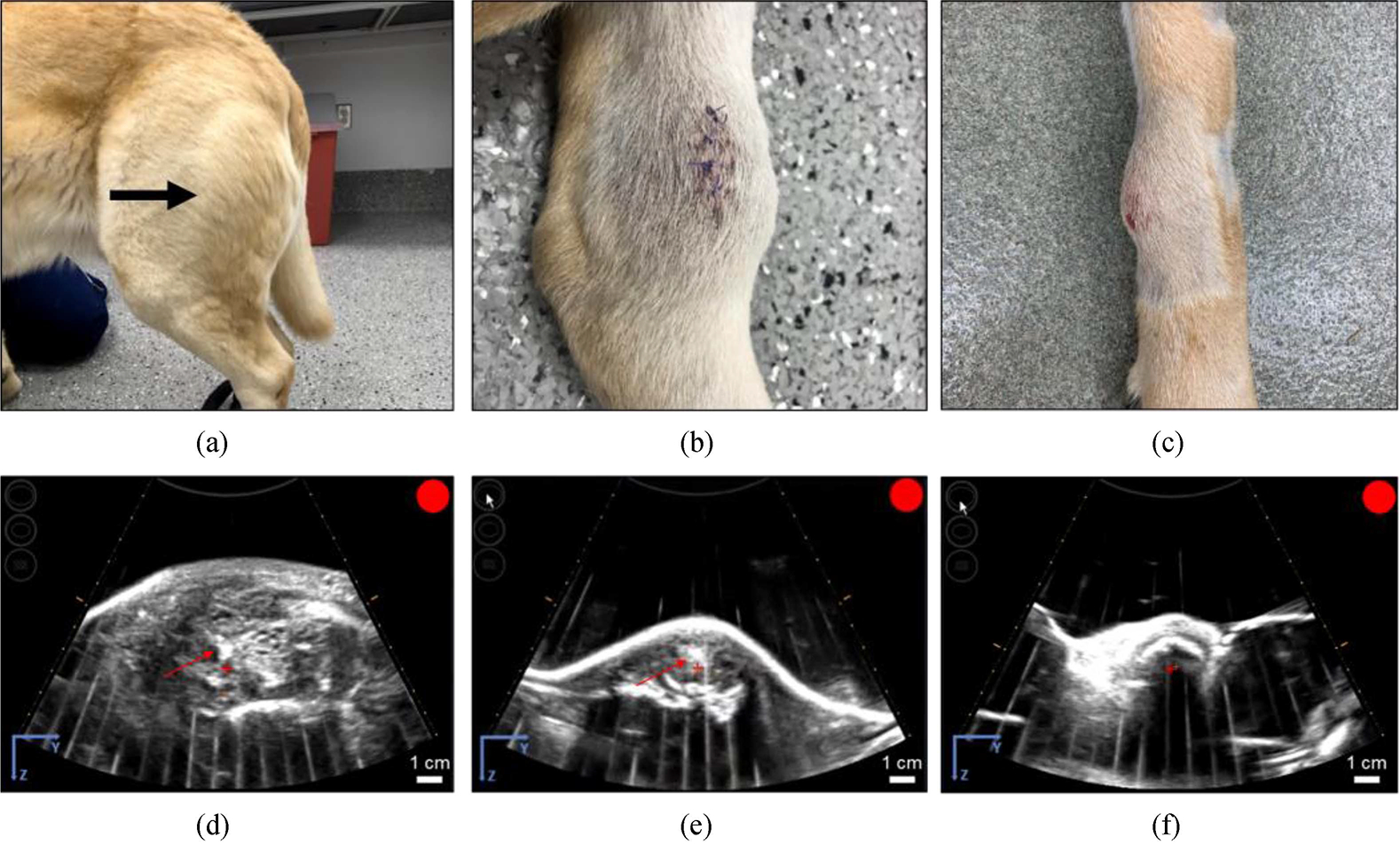
Representative photographs of grossly visible OS tumors and US bubble cloud photos during treatment. (a-c) Patient images showing different gross appearances between the treated primary bone tumors correlating to different radiographic tumor characteristics representing (a) a grossly visible extensive soft tissue component (arrow), (b) a primarily lytic tumor with a small amount of soft tissue component, and (c) a primarily proliferative bone tumor with mostly intact cortical bone. (d-f) B-mode ultrasound images during treatment. Cavitation bubble clouds (arrows) were visible when histotripsy was applied to the soft tissue tumor component and lytic tumors (d,e), but not when applied to patients with proliferative bone tumors and mostly intact cortical bone (f).

Four male castrated dogs and one female spayed dog with a mean age of 8.1 years (±1.96 years) at time of tumor diagnosis were enrolled in the trial [Table I]. All dogs enrolled were purebred dogs, with individual breeds consisting of Labrador retriever (1), golden retriever (1), rottweiler (1), German shepherd (1), and akita (1). Overall tumor composition based on initial radiographic imaging varied amongst patients. Two dogs had primarily proliferative bony lesions, two dogs had primarily lytic bony lesions, and one dog exhibited a more even mix of lytic and proliferative bony lesion. Three dogs also had a substantial soft tissue component to their bone tumor [Table I, Fig. 2].

### B. Histotripsy Treatment

**Fig. 3.**
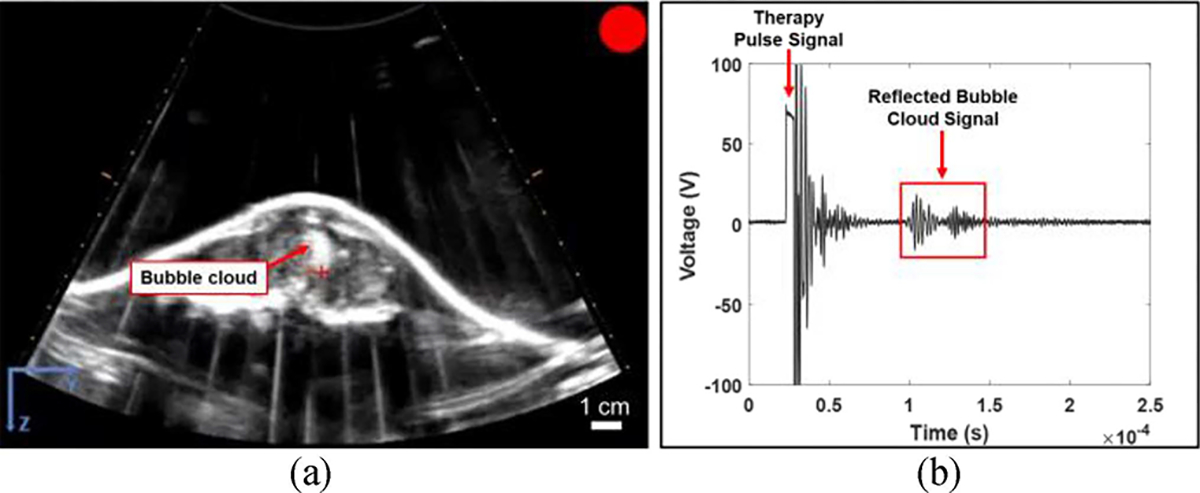
Example PCD waveform collected with a visible bubble cloud. (a) Example bubble cloud (arrow) formed during treatment. (b) Representative PCD waveform collected at the time of treatment shown in (a) with cavitation activity in the waveform boxed.

Generation of clearly visible bubble clouds on real-time ultrasound imaging was achieved in three of the five treatments [Fig. 2(d), (e), Movie S1, Movie S2], but not in the remaining two patients, likely due to ultrasound image artifact(s) caused by bone obstruction [Fig. 2(f), Movie S3]. In these patients, the presence of cavitation was confirmed by PCD signals at the focus similar to the PCD signals measured in the three patients with visible bubble clouds, demonstrating that a bubble cloud was successfully generated at the focus even though it could not be clearly visualized on ultrasound imaging [Fig. 3].

Automated histotripsy treatments were applied at peak negative pressures averaging 29.59 ± 8.17 MPa. Histotripsy treatment duration ranged from approximately 5 to 60 minutes depending on the volume treated. Treatment volumes were centered at depths of 2.1 to 5.6 cm beneath the skin, with points as shallow as 1 cm and as deep as 7 cm targeted during volumetric treatments. In all subjects, cavitation activity was maintained for the duration of the volumetric ablation, as evidenced by ultrasound imaging and/or PCD monitoring. Of the 3 dogs with a visible bubble cloud on ultrasound, the bubble cloud was visible at the focal location of the transducer throughout the entire treatment in two of the three dogs. However, in one dog, visibility of the bubble cloud was consistently lost whenever the focus was moved through one half of the prescribed treatment volume, likely caused by either a heightened cavitation threshold in that section of the tumor or a reduction in the quality of the ultrasound image in these regions due to possible bone obstruction. In addition, during three of the five total treatments, ultrasound imaging and PCD analysis also showed varying degrees of prefocal cavitation at the skin surface.

### C. Adverse Events

No significant adverse events associated with histotripsy were noted. Histotripsy treatment was well-tolerated in all patients, and no clinically significant variation from expected ranges of measured anesthetic parameters were observed. Body temperatures were maintained between 96.0 °F – 101.9 °F. Pulse rates were maintained between 32–78 bpm for 4 patients, with 1 patient ranging between 72–160 bpm. The elevated heart rate of 160 in this subject was noted prior to histotripsy treatment at induction of anesthesia and was attributed to the dog’s anxious temperament. Respiratory rates were maintained between 5–25 breaths per minute in all subjects, and mean blood pressures were maintained between 68–121 mmHg. Oxygen saturation levels were maintained between 96–100%, with an isolated measurement of 93% in 2 patients which were deemed to be clinically insignificant, and potentially spurious. No cardiac arrhythmias were noted under general anesthesia during treatment, and no adverse constitutional clinical signs (lethargy/fatigue, fever, hypothermia) were noted after histotripsy. One dog experienced Grade 1 erythema of the skin at a distal site (outside of the histotripsy treatment path) due to manual pressure from the placement of the bowl of degassed water. This erythema was mild and transient, and resolved by 24 hours after the procedure. All patients underwent standard-of-care limb amputation surgery 18–24 hours post treatment, recovered without complications, and returned home 1–2 days after surgery.

### D. CT With Contrast Imaging Outcomes

**Fig. 4.**
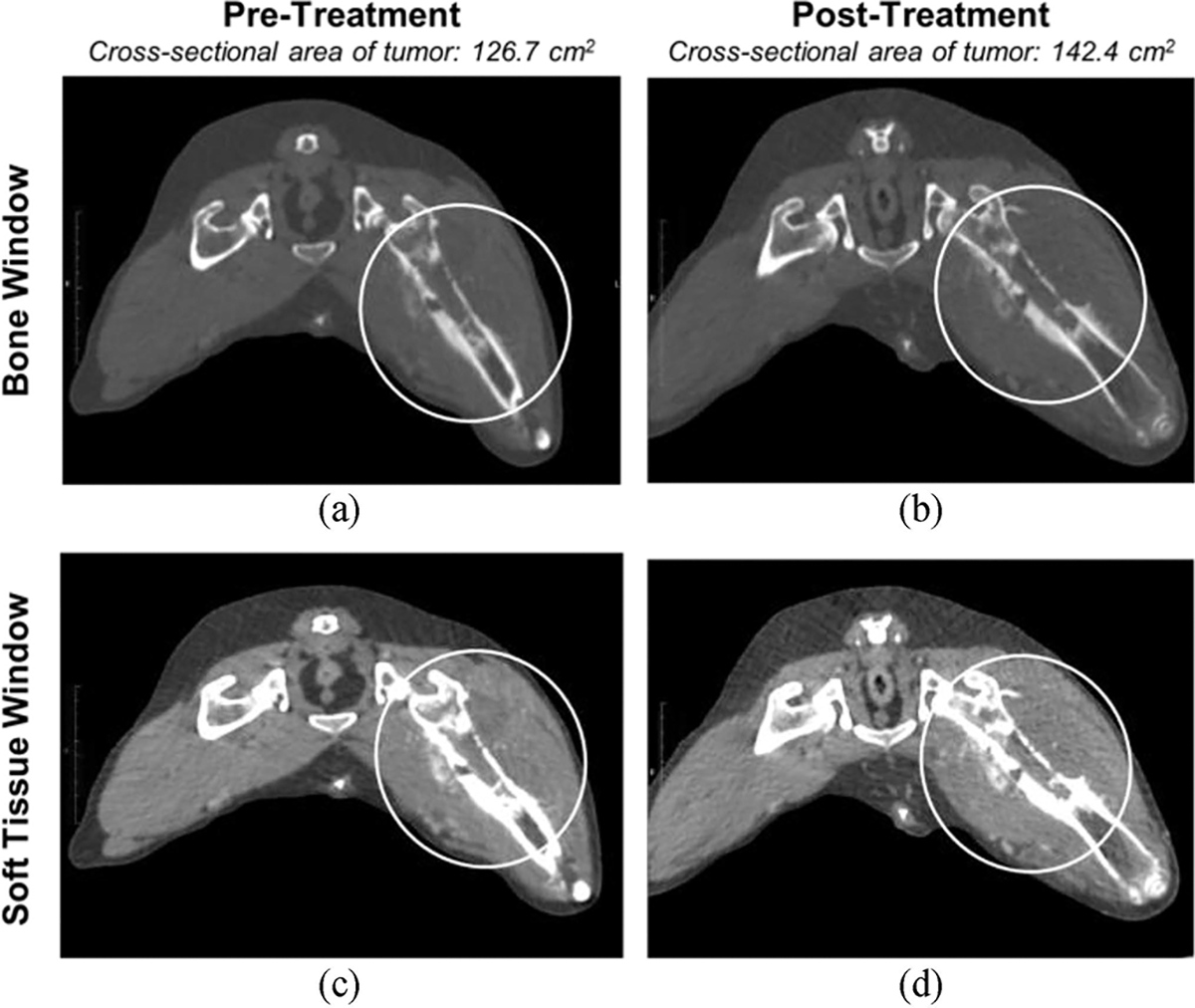
Representative CT images including both bone and soft tissue windows, pre-and post-histotripsy treatment. In both the bone and soft tissue windows, an enlargement of the tumor lesion is observed post-treatment (b,d) compared to pre-treatment (a,c). In each image, the tumor is circled.

Pre- and post-treatment tumor sizes were estimated as cross-sectional measurements based on maximal diameter on multiplanar reformation images [Table II]. Post-treatment CT scans demonstrated no perceivable differences in appearance of bony reaction, pattern of lysis or contrast enhancement within the bony portion of the tumor. General evaluation of pre-and post-treatment CT scans showed enlarged tumor lesions post-treatment, with an average increase of 4.76 ± 5.58 cm^2^ in cross-sectional tumor area following treatment. This finding was not statistically significant (p = 0.163). Due to heterogenicity of tissue attenuation pattern and non-contrasting regions of the soft tissue portions of the tumors, it was not possible to quantify structural changes other than changes in overall size [Fig. 4]. Thus, direct comparisons between ablated and planned volumes could not be made.

### E. Gross and Microscopic Findings

The final histologic diagnoses for the tumors included in this study were osteosarcoma (n = 4) and chondrosarcoma (n = 1). Osteosarcomas were characterized by typical histologic findings of pleomorphic populations of spindle to stellate cells producing variable amounts of osteoid and/or chondroid matrix that was occasionally mineralized. The relative abundance of matrix varied amongst the evaluated patient tumors with some tumors containing higher amounts of matrix and others having larger soft tissue components containing relatively lower amounts of matrix. The chondrosarcoma was composed entirely of neoplastic chondrocytes embedded in a chondroid matrix. Grossly, treatment sites exhibited obvious foci of necrosis characterized by tissue softening, loss, and/or discoloration. Extensive hemorrhage was also prominent. No evidence of histotripsy-induced damage was observed grossly in untreated tissues.

**Fig. 5.**
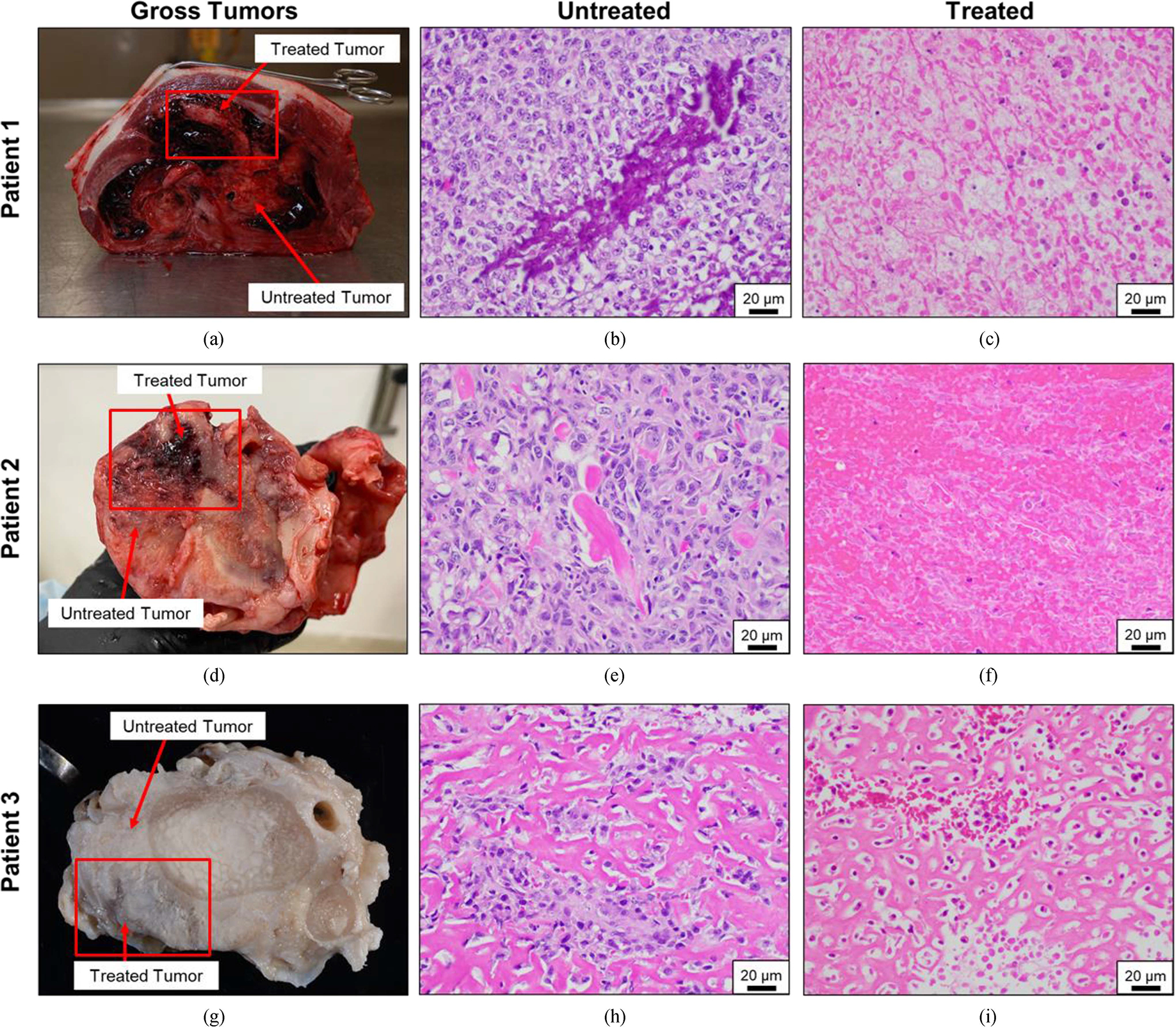
Patient-matched gross pathology and microscopic histology images depicting untreated and treated tumors. (a,d,g) Gross pathology images of primary bone tumors harvested from canine osteosarcoma patients post-limb amputation surgery. Both histotripsy-treated and untreated regions of the tumor are indicated. Images in (a) and (d) were taken immediately following limb amputation surgery before additional tissue processing. The tissue shown in image (g) is formalin-fixed. (b,e,h) Neoplastic osteoblasts, occasionally surrounding bits of osteoid (bright pink staining) (magnification – 40x). (c,f,i) Stained treated tissues exhibited extensive foci of hemorrhage and necrosis, coagulative and lytic cell death, and varying degrees of matrix degeneration (magnification – 40x). Estimated treated tumor regions are boxed in (a), (d), (g).

Microscopically, all treated areas exhibited similar features in varying amounts. All treatment sites, regardless of degree of osteoid matrix, exhibited foci of acute hemorrhage as well as a combination of lytic and coagulative necrosis [Fig. 5(f), (i)]. Lytic necrosis was characterized by loss of recognizable cell architecture and replacement by cellular debris [Fig. 5(c), (f)]. Coagulative necrosis was characterized by preservation of cell architecture but cells exhibit increased eosinophilic staining (pinkness), angular borders, and variably condensed nuclei and was used to describe cells that were rendered non-viable by histotripsy treatment but not fully lysed [Fig. 5(i)]. In treated areas, there was variable degeneration of both osteoid and chondroid matrix associated with decreased cellularity and increased eosinophilic staining (pinkness). Both acute and coagulative necrosis could occasionally be appreciated in untreated samples from the same tumor, however, untreated areas generally lacked the acute hemorrhage and foci of necrosis were far less extensive. Microscopically, tumor ablation was noted throughout the region of planned histotripsy ablation. In some cases, foci of viable cells remained within targeted treatment regions.

**Fig. 6.**
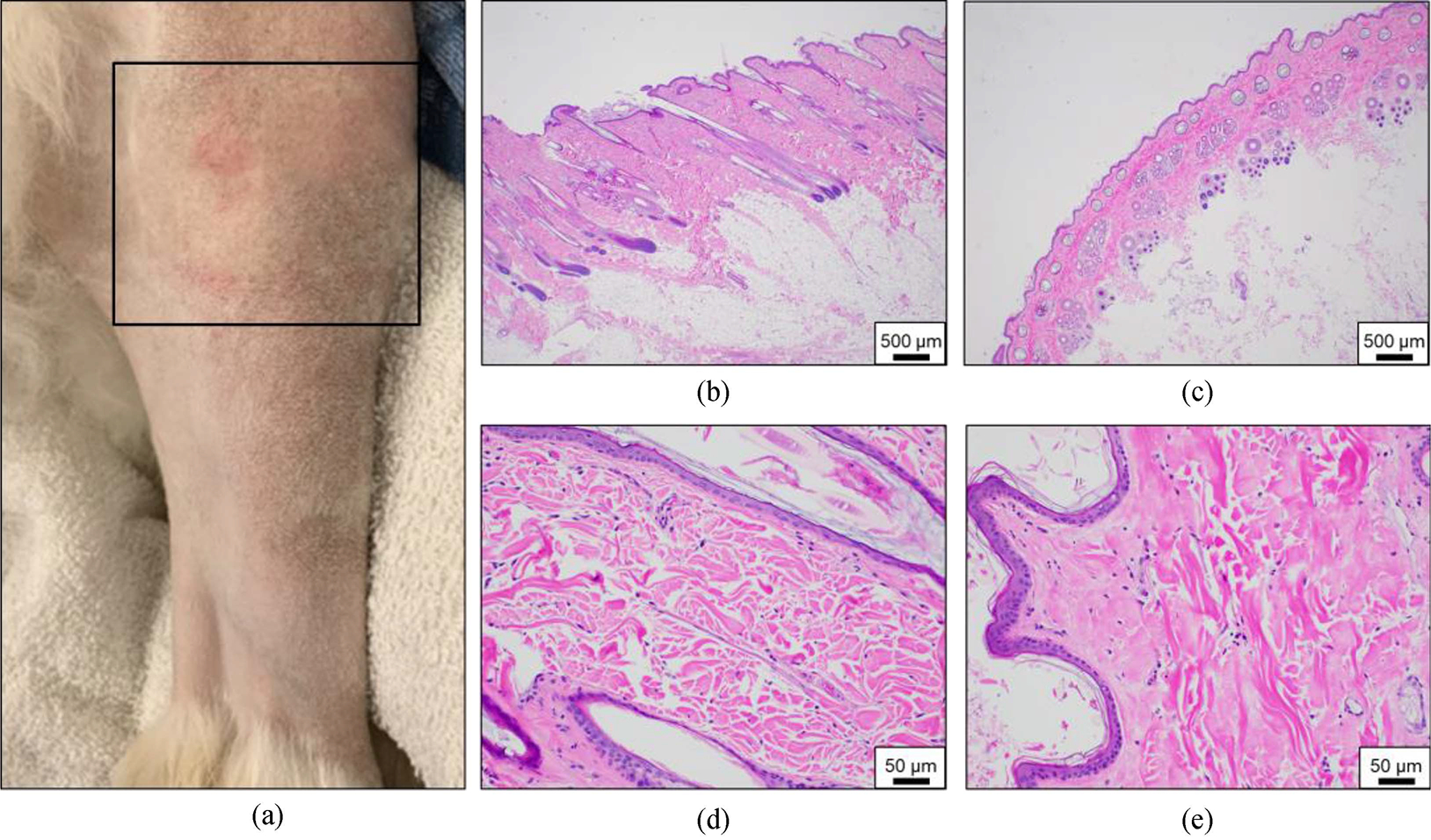
Gross and microscopic evaluation of the skin overlying targeted tumor tissues. (a) Photograph of the skin immediately post-treatment overlying the tumor (treatment area boxed). (b,d) Histologically typical skin (b – magnification, 2x, d – magnification, 20x). (c,e) Skin overlying the histotripsy-treated area showing hyalinization of dermal collagen (c – magnification, 2x, e – magnification, 20x).

No gross evidence of damage to the skin was identified in the areas overlying the treated tumor. However, in two of five treated patients, there was evidence of microscopic abnormalities. In one patient, small amounts of inflammation, including aggregates of neutrophils and macrophages, were identified in the dermis overlying the treatment area. It is unlikely that the abnormalities in this patient were due to histotripsy treatment since other sections of skin taken from untreated areas exhibited similar inflammation along with ruptured hair follicles. In the second patient with microscopic skin damage, hyalinization of dermal collagen was identified in the skin in the histotripsy treatment path, but not in untreated regions [Fig. 6]. While noteworthy due to the investigative nature of the study, the clinical significance associated with the degree of collagen hyalinization observed in this patient is questionable. No gross evidence of skin damage was observed in this patient nor were lesions such as necrosis and hemorrhage identified to support true tissue damage. Additionally, no signs of thermal injury were observed within the treatment volume or overlying tissues in any of the patients.

### F. Immune Response Evaluation

**Fig. 7.**
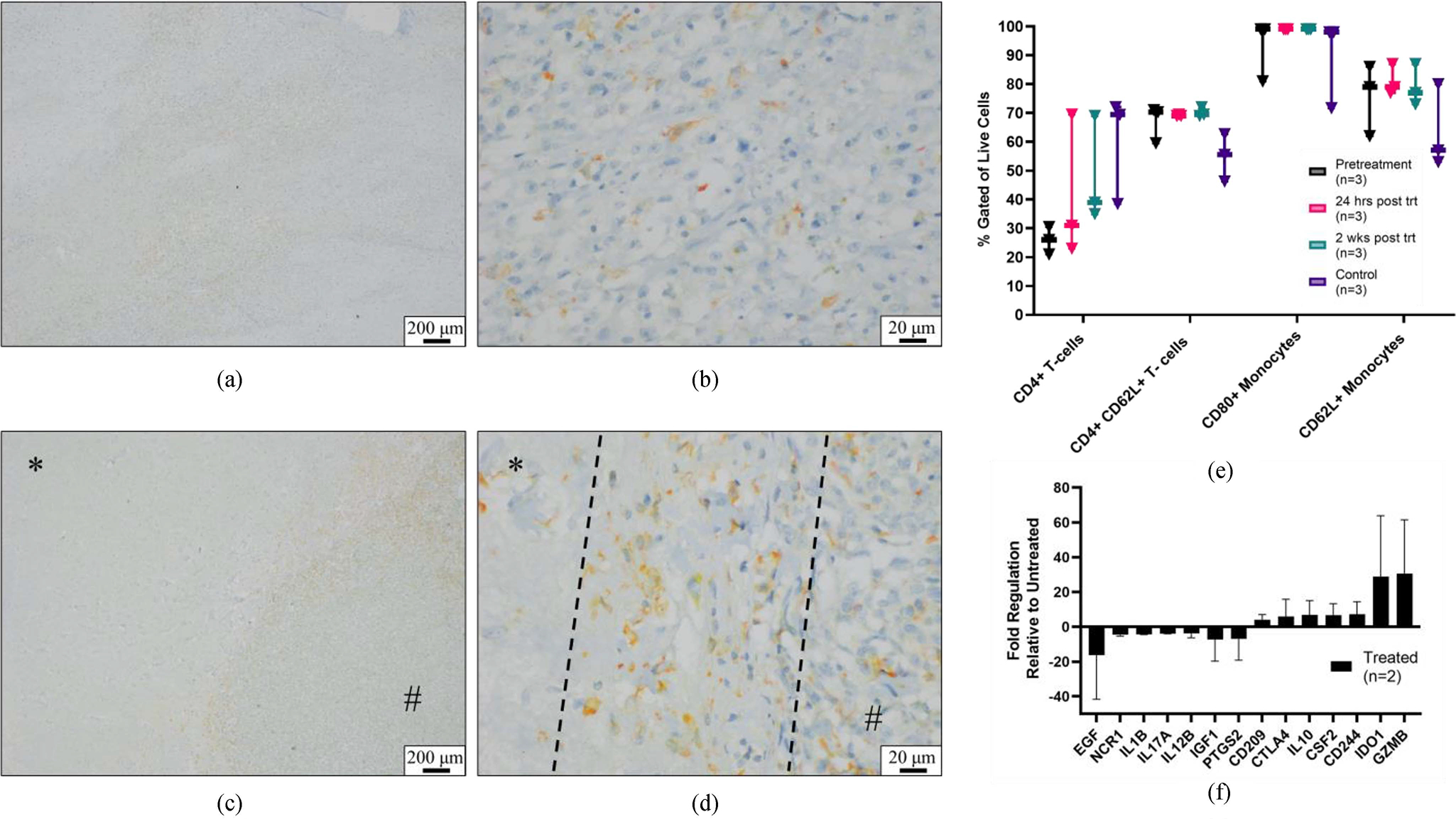
Immune response evaluation. (a-d) Representative chromogenic multiplex IHC images of macrophage populations taken from patient 2. (a,b) In untreated tumor samples, CD206 and IBA-1 double positive stained cells (orange) were seen loosely distributed throughout intact tumor tissue. (c,d) Concentrated areas of CD206 and IBA-1 double positive stained macrophages at the treatment interface. Ablated tumor tissue (*) and intact tumor tissue (#), and in (d) the treatment interface is denoted by dashed lines. (magnification: a,c – 4x and b,d – 40x). (e) Flow cytometry results of PBMCs analyzed pretreatment, 24 hours, and 2 weeks post histotripsy treatment. For analysis, monocytes = CD11b+ CD14+ live cells corresponding to monocyte size and granularity (forward scatter vs. side scatter analysis), and T-cells = live cells corresponding to lymphocyte size and granularity (forward vs. side scatter analysis). (f) Gene expression changes in treated tumor samples relative to paired untreated tumor samples (n=2). Error bars are standard deviation.

Multiplex IHC evaluation revealed that in both treated and untreated regions of the tumor samples, immune cell populations were generally of similar composition. Macrophage assessment (IBA-1, CD206, and iNOS) showed relatively high numbers of individualized, IBA-1 and CD206 double positive stained cells scattered throughout intact untreated tumor cells [Fig. 7(a), (b)]. In one sample, the treatment interface was evaluated and concentrated regions of IBA-1 and CD206 double positive stained cells were noted at the interface [Fig. 7(c), (d)]. Assessment of tumor samples for lymphocyte infiltration (CD3, CD4, CD8, and CD79a) revealed no distinguishable differences between treated and untreated samples. There were no observable differences in macrophage or lymphocyte populations in the control dog samples when compared to study samples of untreated tumor.

Flow cytometry analysis of circulating immune cells showed a mean increase in CD4+ T-cells 24 hours and 2 weeks after histotripsy treatment compared to pretreatment. On average, healthy control dogs had a greater percentage of CD4+ T-cells compared to OS patients but decreased CD62L expression in the CD4- T cell and monocyte populations. No changes in CD80 monocyte expression were observed [Fig. 7(e)].

Of the 5 enrolled patients, 2 had tumor samples that contained satisfactory RNA quality for analysis. Of the 89 evaluated genes, 14 had a fold change value of 3-fold or greater. Functional gene enrichment analysis revealed that these 14 genes were associated with gene ontology biological pathways immune and inflammatory responses, regulation of cell death, and the KEGG pathway natural killer cell mediated cytotoxicity [Supplemental Table II].

## IV. Discussion

In this study, histotripsy bubble clouds were successfully generated *in vivo* within the tumors of five dogs with suspected osteosarcoma, resulting in the precise and non-invasive ablation of the targeted tumor volumes. The findings of this study provide initial evidence showing that histotripsy can be employed as a new method for the precise, non-invasive, and non-thermal ablation of bone tumors. During all treatments, real-time ultrasound imaging and/or PCD monitoring showed that focal histotripsy bubble clouds were formed at the targeted points within the tumor, with no off-target cavitation activity measured. Post-treatment evaluations revealed macroscopic regions of hemorrhage and necrosis in targeted areas, microscopically characterized by a loss of viable tumor cells and varying degrees of hemorrhage and matrix degeneration. No off-target histotripsy damage was identified in untreated tissues, and cross-sectional gross measurements of the ablated tumor regions were fully contained within the planned treatment volumes. Notably, destruction of tumor cells was achieved even in highly mineralized areas, with only acellular matrix left behind after treatment. In all five dogs treated, the histotripsy procedure was well-tolerated and patient safety was not jeopardized, with no clinically significant adverse events observed.

While previous reports have demonstrated the feasibility of *in vivo* histotripsy treatment for multiple applications, including treatments within the proximity of bone [36], [37], this is the first study to investigate the *in vivo* feasibility of ablating primary bone tumors with histotripsy. This application represents unique challenges for histotripsy compared to soft tissue treatments due to the large amount of ultrasound attenuation caused by bone and the impact of tissue mechanical properties on the histotripsy bubble cloud behavior and ablation. Prior studies have shown that histotripsy can non-invasively generate precise bubble clouds through transcostal and transcranial acoustic windows [36], [38]. However, the proximity of bone so close to the treatment focus has the potential to make it more difficult to generate precise cavitation bubble clouds when targeting bone tumors. In addition, prior work has shown that tissues with more fibrous and calcified components are more difficult to treat with histotripsy [29], [39], representing a further challenge for the successful treatment of bone tumors with histotripsy.

Comparison of pre- and post-treatment CT scans revealed enlargement of the treated tumor region, potentially due to transient inflammatory swelling associated with histotripsy ablation. Although histological analysis showed tumor ablation throughout the targeted region of the tumor, extensive and detailed changes relating to histotripsy ablation were difficult to characterize on CT due to the heterogenicity of the attenuation pattern in the soft tissue regions, the variation in tumor shape, and contrast enhancement pattern. Additionally, variations in tumor shape and location limited our ability to standardize an image plane for acquiring exact tumor measurements, resulting in intra- and inter-patient variation. These findings suggest that future studies should explore other imaging modalities to evaluate histotripsy treatment in OS patients, such as MRI, which has previously been used to image tissue changes after histotripsy and may offer advantages over CT [24], [40].MRI is not a routine imaging modality for staging canine OS patients, but it is the standard imaging modality for staging and monitoring human OS patients [41]. Our group has recently utilized MRI for histotripsy treatment evaluation pre- and post-treatment in preclinical orthotopic OS murine models, and ablation of tumor tissue was clearly delineated on MR images (unpublished data). This observation is also supported by previous histotripsy liver ablation studies in pigs and data from a recent human clinical trial for histotripsy ablation of liver tumors which used MRI as the primary modality for assessing ablation effects after treatment [24], [40]. Since acquiring optimal images pre- and post-histotripsy is essential for monitoring treatment success and developing patient-specific treatment plans, future studies evaluating the efficacy of MRI for assessing histotripsy-treated OS lesions are warranted.

Another factor that should be explored in future studies is the impact of tumor characteristics including size, location, and composition on histotripsy ablation. Tumor characteristics varied amongst the five patients treated in this study and were likely the cause of the inability to clearly visualize the histotripsy bubble cloud in three of the five patients. For instance, larger tumors near the skin surface and tumors with an extensive soft tissue component were more easily discernable on ultrasound imaging. In contrast, treatment targeting and bubble cloud visualization were more difficult for deeper tumors located in proximal regions of the appendicular skeleton, such as the proximal humerus or femur. Similarly, tumors with primarily proliferative bone or those located within the medullary cavity with minimal cortical bone lysis were more difficult to visualize and target due to imaging artifacts (i.e., shadowing) caused by the presence of bone. These imaging artifacts impeded device alignment and treatment monitoring using conventional B-mode ultrasound imaging methods. In the current study, these limitations were overcome by utilizing anatomical landmarks and pre-treatment CT images in order to target the desired regions. In addition, cavitation activity was monitored in these subjects by using one of the elements on the histotripsy transducer for passive cavitation detection (PCD) in order to monitor the backscatter signal from cavitation bubbles formed at or near the histotripsy transducer’s focus [30], [42]. Suspected focal cavitation activity was observed on the PCD signal for all treatments, including cases with and without visible bubble clouds observed on ultrasound imaging. Future work could expand on this approach in order to overcome the targeting limitations for OS tumors encased in cortical bone by using 3D cavitation mapping approaches that have previously been developed for transcranial histotripsy applications [42]. Alternatively, another option that could be explored is the use of MRI guidance and imaging feedback methods [43], [44].

Despite the imaging challenges encountered, the results of this study showed that histotripsy was able to generate effective ablation in the targeted regions for all five patients, regardless of tissue composition or bubble cloud visibility. One of the treated tumors was ultimately diagnosed as a chondrosarcoma, suggesting that histotripsy may be a viable ablation technique for canine chondrosarcoma. A combination of gross and microscopic histological analyses showed that the treated tissues were grossly and histologically distinguishable from untreated tissues. Hemorrhage and tissue necrosis were observed grossly, and zones of increased hemorrhage, loss of viable tumor cells, and destruction of cellular architecture were noted histologically. In some cases, foci of viable cells remained within portions of the treated tumor regions, suggesting that the employed treatment dose of 500 pulses per point was not always sufficient to achieve complete ablation in all regions of the OS tumors targeted in this study. This dose is notably lower than the treatment dosage of 4000 pulses per point employed in our prior *ex vivo* excised bone tumor study [25] and was selected to reduce the treatment time for canine patients. As a result, future work is warranted to optimize the histotripsy parameters and treatment doses required to achieve complete and efficient ablation of OS tumors of all compositions and to develop patient-specific treatment plans that address the heterogeneity in the composition of bone tumors. Future work should also ensure that there are no complications due to off-target cavitation when treating OS tumors, particularly at the skin’s surface or the bone interface. Although the minor skin abnormalities observed histologically in this study were not determined to be clinically significant, they highlight a potential risk of using histotripsy to ablate superficial tumors.

The OS tumor microenvironment (TME), like other cancers, is immunosuppressive, allowing cancer cells to evade the immune response and proliferate and form malignant tumors [45], [46]. We hypothesized that histotripsy treatment would modulate the immunosuppressive OS TME and stimulate a proinflammatory anti-tumor immune response, which has previously been reported to be associated with histotripsy treatment [47]–[50]. The stimulation of a pro-inflammatory immune response would lead to infiltration of pro-inflammatory macrophages, characterized by double positive staining of IBA1 and iNOS and potentially an infiltration of lymphocytes. However, we observed an increase in macrophages of the repair phenotype, characterized by double positive staining for IBA1 and CD206, and no differences in lymphocyte populations between untreated and treated regions of the tumor. Furthermore, we observed an increase in proportions of systemic CD4+ T lymphocytes after histotripsy ablation but did not observe any changes in monocyte population within our small sample population. It is likely that the small sample population and limited sampling timepoints restricted the findings of this study, and additional studies with larger patient populations and additional evaluation timepoints are needed to further investigate the immunological responses associated with histotripsy treatment for OS. Overall, our results suggest potential systemic and intratumoral immune stimulation in response to histotripsy, but our observations are preliminary and limited to a small group of dogs.

This report details a treat-and-resect feasibility study designed to evaluate whether histotripsy could effectively and safely ablate OS tumors *in vivo* in canine OS patients still receiving current standard of care amputation. Future work is needed to evaluate the long-term safety and efficacy of histotripsy for this application. These future studies should investigate the feasibility of ablating the entire OS tumor volume to achieve negative margins and further explore the immunogenic potential of histotripsy [22], [24], [48], [49], [51].

## V. Conclusion

This initial *in vivo* feasibility study demonstrates that histotripsy has the potential to be utilized for the non-invasive ablation of bone tumors while also highlighting some of the challenges of using histotripsy for this application. The results of this study showed effective histotripsy ablation was achieved in targeted regions of all treated tumors without clinically significant adverse effects or off-target damage to untreated tissues. Overall, this work suggests that histotripsy is a promising therapy for the non-invasive treatment of primary bone tumors in dogs and should be further investigated for this application in both veterinary and human medicine.

## Supplementary Material

supp2-3191069

supp3-3191069

supp4-3191069

supp5-3191069

supp1-3191069

## Figures and Tables

**TABLE I:** Patient Demographics and Tumor Characteristics

Patient	Breed	Age (years)	Tumor Location	Histologic Diagnosis	Radiographic Characteristics

1	Yellow Lab	12	Proximal Femur	Osteosarcoma	Lytic Bone and Soft Tissue Component
2	Akita	7.5	Distal Tibia	Osteosarcoma	Mix of Lytic and Proliferative Bone and Soft Tissue Component
3	German Shepherd	7	Distal Radius	Osteosarcoma	Proliferative Bone
4	Golden Retriever	7	Proximal Tibia	Osteosarcoma	Proliferative Bone
5	Rottweiler	7	Proximal Humerus	Chondrosarcoma	Lytic Bone and Soft Tissue Component

Five canine patients (n = 4 osteosarcoma, n = 1 chondrosarcoma) received partial tumor ablations. Tumor composition varied between patients, ranging from soft tissue and lytic bone to proliferative neoplastic bone.

**TABLE II:** Comparison of Tumor Size Pre- and Post-Treatment

Patient	Pre-treatment Tumor Size (cm^2^)	Post-treatment Tumor Size (cm^2^)	Difference (cm^2^) (Post vs. Pre-treatment)

1	126.687	142.378	15.691
2	17.281	20.369	3.088
3	3.729	3.937	0.208
4	14.782	16.287	1.505
5	51.219	54.519	3.300

Pre- and post-treatment tumor sizes estimated as cross-sectional measurements based on maximal diameter on multiplanar reformation images. Results showed enlarged tumor lesions post-treatment, possibly due to histotripsy-induced swelling.
