# Supplementary material for: Histotripsy Ablation of Spontaneously Occurring Canine Bone Tumors In Vivo
Source: IEEE Trans Biomed Eng. Author manuscript; Available in PMC 2024 Jan 14. (PMC9921194; doi:10.1109/TBME.2022.3191069)
Supplement: supp2-3191069 [file NIHMS1860980-supplement-supp2-3191069.docx]

SUPPLEMENTAL TABLE II

Functional Gene Enrichment Analysis Pathways

| Functional Gene Enrichment Analysis | Genes |
| --- | --- |
| GO: BP - Immune Response | IL1B, IL12B, CTLA4, IL10, CSF2, CD244, and GZMB |
| GO: BP - Inflammatory Response | IL1B, IL12B, IGF1, PTGS2, and IL10 |
| GO: BP - Regulation of Cell Death | IL1B, IL12B, IGF1, PTGS2, CTLA4, IL10, and CSF2 |
| KEGG Pathway - Natural Killer Cell Mediated Cytotoxicity | NCR1, CSF2, CD244, and GZMB |

Analyzed genes with a ≤ -3 or ≥ 3-fold change were included for the analysis. GO = Gene Ontology, BP = Biological Pathway, KEGG = Kyto Encyclopedia of Genes and Genomes.
