# Supplementary material for: Histotripsy Ablation of Spontaneously Occurring Canine Bone Tumors In Vivo
Source: IEEE Trans Biomed Eng. Author manuscript; Available in PMC 2024 Jan 14. (PMC9921194; doi:10.1109/TBME.2022.3191069)
Supplement: supp1-3191069 [file NIHMS1860980-supplement-supp1-3191069.docx]

SUPPLEMENTAL TABLE I

Canine Immunity And Inflammation Crosstalk Array Genes

| Symbol | Description | Symbol | Description |
| --- | --- | --- | --- |
| ACKR3 | Chemokine (C-X-C motif) receptor 7 | IL13 | Interleukin 13 |
| AICDA | Activation-induced cytidine deaminase | IL15 | Interleukin 15 |
| BCL2 | B-cell CLL/lymphoma 2 | IL17A | Interleukin 17A |
| BCL2L1 | BCL2-like 1 | IL1A | Interleukin 1, alpha |
| CCL2 | Chemokine (C-C motif) ligand 2 | IL1B | Interleukin 1, beta |
| CCL20 | Chemokine (C-C motif) ligand 20 | IL1R1 | Interleukin 1 receptor, type I |
| CCL28 | Chemokine (C-C motif) ligand 28 | IL2 | Interleukin 2 |
| CCL4 | Chemokine (C-C motif) ligand 4 | IL22 | Interleukin 22 |
| CCL5 | Chemokine (C-C motif) ligand 5 | IL23A | Interleukin 23, alpha subunit p19 |
| CCR1 | Chemokine (C-C motif) receptor 1 | IL4 | Interleukin 4 |
| CCR10 | Chemokine (C-C motif) receptor 10 | IL5 | Interleukin 5 (colony-stimulating factor, eosinophil) |
| CCR2 | C-C motif chemokine receptor 2 | IL6 | Interleukin 6 (interferon, beta 2) |
| CCR4 | Chemokine (C-C motif) receptor 4 | IRF1 | Interferon regulatory factor 1 |
| CCR5 | Chemokine (C-C motif) receptor 5 | MYC | V-myc myelocytomatosis viral oncogene homolog (avian) |
| CCR7 | Chemokine (C-C motif) receptor 7 | MYD88 | Myeloid differentiation primary response gene (88) |
| CCR9 | Chemokine (C-C motif) receptor 9 | NFKB1 | Nuclear factor of kappa light polypeptide gene enhancer in B-cells 1 |
| CD274 | CD274 molecule | NOS2 | Nitric oxide synthase 2, inducible |
| CSF1 | Colony stimulating factor 1 (macrophage) | PDCD1 | Programmed cell death 1 |
| CSF2 | Colony stimulating factor 2 (granulocyte-macrophage) | PTGS2 | Prostaglandin-endoperoxide synthase 2 (prostaglandin G/H synthase and cyclooxygenase) |
| CSF3 | Colony stimulating factor 3 (granulocyte) | SPP1 | Secreted phosphoprotein 1 |
| CTLA4 | Cytotoxic T-lymphocyte-associated protein 4 | STAT1 | Signal transducer and activator of transcription 1, 91kDa |
| CXCL10 | Chemokine (C-X-C motif) ligand 10 | STAT3 | Signal transducer and activator of transcription 3 (acute-phase response factor) |
| CXCL11 | Chemokine (C-X-C motif) ligand 11 | TGFB1 | Transforming growth factor, beta 1 |
| CXCL12 | Chemokine (C-X-C motif) ligand 12 | LAMP1 | Toll-like receptor 2 |
| CXCL5 | Chemokine (C-X-C motif) ligand 5 | TLR2 |  |
| CXCR1 | Chemokine (C-X-C motif) receptor 1 | TLR3 | Toll-like receptor 3 |
| CXCR2 | Interleukin 8 receptor, beta | TLR4 | Toll-like receptor 4 |
| CXCR3 | Chemokine (C-X-C motif) receptor 3 | TLR7 | Toll-like receptor 7 |
| CXCR4 | Chemokine (C-X-C motif) receptor 4 | TLR9 | Toll-like receptor 9 |
| CXCR5 | Chemokine (C-X-C motif) receptor 5 | TNF | Tumor necrosis factor |
| EGF | Epidermal growth factor | IL18 | Interleukin 18 (interferon-gamma-inducing factor) |
| EGFR | Epidermal growth factor receptor | FASLG | Fas ligand (TNF superfamily, member 6) |
| FOXP3 | Forkhead box P3 | CXCL8 | Interleukin 8 |
| GZMA | Granzyme A (granzyme 1, cytotoxic T-lymphocyte-associated serine esterase 3) | APLNR | Apelin receptor |
| GZMB | Granzyme B | JAK1 | Janus kinase 1 |
| HIF1A | Hypoxia inducible factor 1, alpha subunit (basic helix-loop-helix transcription factor) | JAK2 | Janus kinase 2 |
| IDO1 | Indoleamine 2,3-dioxygenase 1 | ICAM1 | Intercellular adhesion molecule 1 |
| IFNG | Interferon gamma | IL2RA | Interleukin 2 receptor, alpha |
| IGF1 | Insulin-like growth factor 1 (somatomedin C) | CD244 | CD244 molecule, natural killer cell receptor 2B4 |
| IL10 | Interleukin 10 | CCL13 | Chemokine (C-C motif) ligand 13 |
| IL12A | Interleukin 12A (natural killer cell stimulatory factor 1, cytotoxic lymphocyte maturation factor 1, p35) | CD209 | CD209 molecule |
| IL12B | Interleukin 12B (natural killer cell stimulatory factor 2, cytotoxic lymphocyte maturation factor 2, p40) | TBX21 | T-box 21 |

A table of all genes included in the Qiagen RT2 Canine Immunity and Inflammation Crosstalk Array.
